# Human Activity and Motion Pattern Recognition within Indoor Environment Using Convolutional Neural Networks Clustering and Naive Bayes Classification Algorithms

**DOI:** 10.3390/s22031016

**Published:** 2022-01-28

**Authors:** Ashraf Ali, Weam Samara, Doaa Alhaddad, Andrew Ware, Omar A. Saraereh

**Affiliations:** 1 Department of Electrical Engineering, Faculty of Engineering, The Hashemite University, Zarqa 13133, Jordan; eloas2@hu.edu.jo; 2 Estarta Co., Ltd., Amman 11942, Jordan; weaamsamara@gmail.com (W.S.); doaa.alhaddad97@gmail.com (D.A.); 3 Faculty of Computing, Engineering and Sciences, University of South Wales, Pontypridd CF37 1DL, UK; andrew.ware@southwales.ac.uk

**Keywords:** human activity recognition, CNN, sensors, machine learning, motion pattern, Naive Bayes

## Abstract

Human Activity Recognition (HAR) systems are designed to read sensor data and analyse it to classify any detected movement and respond accordingly. However, there is a need for more responsive and near real-time systems to distinguish between false and true alarms. To accurately determine alarm triggers, the motion pattern of legitimate users need to be stored over a certain period and used to train the system to recognise features associated with their movements. This training process is followed by a testing cycle that uses actual data of different patterns of activity that are either similar or different to the training data set. This paper evaluates the use of a combined Convolutional Neural Network (CNN) and Naive Bayes for accuracy and robustness to correctly identify true alarm triggers in the form of a buzzer sound for example. It shows that pattern recognition can be achieved using either of the two approaches, even when a partial motion pattern is derived as a subset out of a full-motion path.

## 1. Introduction

Modern smart intruder alarm and Human Activity Recognition (HAR) systems consist of networks of integrated electronic devices and sensors connected to a centralised control unit to protect against intruders by distinguishing between legitimate and illegitimate activity. In contrast to conventional security systems that respond to a single sensor trigger, the intelligent system uses machine learning techniques and IoT (Internet of Things) sensor infrastructure to detect and classify complex motion patterns. Different types of sensors (such as the Passive Infrared Sensor PIR, motion sensor and the ultrasonic sensor) are often used to detect different movement patterns. Similarly, Human Activity Recognition (HAR) uses the same sensors and rely on deep data analysis and machine learning algorithms to classify the movement based on the application needed, this concept can by deployed in many practical applications, such as early diagnosis of human ageing symptoms with dementia, and can also be used to define anomalies in employee transitions or motions within an indoor environment, as well as as part of burglary alarm systems that detect anomalies in human behaviour.

Regardless of the precise definition of the application, sensors collect data of activity within a predefined environment, time, and space. Activity learning techniques use this data to identify normal, routine motion in terms of forecasted activities. Then, when an abnormal movement is detected, the system will trigger an alarm, notification, or reaction mechanism. Learning activities provide a better data source for the smart alarm system and improve security [1]. The human activity recognition mechanism depends on motion pattern detection, it makes use of efficient algorithms that can process significant quantities of data. Additionally, the algorithms can classify, in near real-time, the detected motion as routine or malicious behaviour. Activity recognition algorithms depend on data collected using the sensors in the home. Daily activities such as entering or exiting the building, waking up or sleeping routine, moving in the kitchen and corridors, depend on the layout of the building, obstacles, and human behaviour. Activity recognition has some unique machine learning challenges, for example, sometimes the data does not indicate the type of activity. Activity recognition consists of several steps including raw sensor data collection, data pre-processing and segmentation, feature extraction, and supervised machine learning. The motion sensor’s raw data has a binary output (movement detected or not). However, the time duration of a particular state (for example, measuring how long a person stays in a room) is also an important measure. The machine learning models used for activity recognition vary by type of sensor [2]. In the prototype, motion sensors are used in favour of cameras as it provides more privacy for legitimate activity and can also function in different ambient light and visibility conditions [3].

The developed prototype in this research only considers activity recognition for a single person in an indoor environment. While the prototype can model normal motion for the residents of real smart homes, synthetic but realistic data can also be generated that reflects real-world motion patterns [1]. Two classes of data pattern are needed to reflect either normal or abnormal behaviours and can be used for both testing and training purposes for any supervised learning algorithm. Therefore, in such smart systems, false positives (raising an alarm needlessly) and false negatives (not raising the alarm when there is a confirmed anomaly in the protected space) are both significant issues. The accuracy of such a system is critical, for example, classifying a suspicious motion as a normal motion is considered more critical than classifying a normal motion as abnormal [3].

Following the literature presented before, it becomes clear that using another approach that combine two machine learning techniques will improve the performance of the system. A Convolutional Neural Network (CNN) has been shown to offer a high accuracy rate if applied over two dimensional spatial data [4,5,6]. The CNN is effective for image recognition tasks [7]. Whereas in [8], the authors proposed a Shared Hidden Layer Deep Convolutional Neural Network (SHL-CNN) for image character recognition. The authors showed that the SHL-CNN can reduce recognition errors by 16–30% compared with models trained by characters of only one language using conventional CNN, and by 35.7% compared with state-of-the-art methods. Thus, this approach is combined with the Naive Bayes classifier using living room path images detected using ultrasonic sensors to improve the accuracy and performance of the prototype system. We can summarise the contributions of this paper as follows:

- Combining two machine learning approaches (Naive Bayes and CNN) to increase the detection accuracy;
- Deploying a CNN in a room, represented by the prototype, without the need for expensive and privacy violating equipment (i.e., cameras). Instead, an array of ultrasonic sensors are used and data are processed later to reflect the path pattern image;
- Exploit the Hidden Markov Model (HMM) features for the Naive Bayes classifier to exploit simplicity of the classifier over the binary signals of PIR sensors;
- Classify the motion patterns for both full tracks (starting from the entrance till the exit) or even via having partial track information via extracting features with an acceptable accuracy rate.

The rest of the paper is organised as follows: Related work will be summarised in Section 2, and the methodology of the research along with the adopted algorithm and selected tools and prototype will be presented in Section 3. Results and further discussion will be presented in Section 4. A mathematical description of the motion pattern will be explained in Section 5, and conclusions presented in Section 6.

## 2. Related Work

In terms of sensors, to harvest the required data, researchers have investigated the use of accelerometers (wearable sensors) for recognising activities such as walking, running, sitting, and standing [3,9,10]. Others have used smart phones equipped with accelerometers to recognise such activities [9]. Moreover, researchers have used accelerometers to detect human motion based on 3-dimensional inertial sensor data [11]. In addition to accelerometers, environment sensors such as infrared-based motion detectors, door sensors, and temperature sensors have been used for an activity recognition purpose [12,13] and for investigation security for smart homes based on activity learning techniques [1]. Video camera surveillance is also common in security applications [14,15]. However, such surveillance has raised privacy concerns [12]. In the prototype developed in this research, the motion pattern between different rooms is detected using a Passive Infrared sensor (PIR) that can detect human motion and an ultrasonic sensor that can detect an individual’s exact location. The collected data of the two sensor types categories are integrated and utilised by the machine learning algorithm for the highest accuracy and least computational complexity.

The input data collected by the sensors, which is often sequential and noisy, must pass through several steps: Pre-processing the raw data, dividing it into subsets of a certain window size, and then extracting features to use in the classification process [1,10]. The correct selection of features will help improve the performance of classification and recognition [9]. Researches have applied many different types of data pre-processing and feature extraction methods with varying degrees of success [11,12,13,16,17,18,19,20]. In terms of window size, some researchers have opted for a fixed window size [3,18], while others have used a varying window size [19]. In contrast, [11,12] relied on a combination of pre-processing and feature extraction methods.

Different pre-processing methods that rely on statistical and physical features extraction algorithms can be applied over raw sensor data, for example using four features, that are analysed statistically over time, frequency, and time frequency domain methods, which may result in an average performance accuracy of 90.89% for the best combination of pre-processing and feature extraction methods [11]. While in some experiments it was observed that combining a fixed length Sensor Window with Mutual Information-based (SWMI) weighing of sensor events and adding two levels of a fixed length sensor window approach that includes the probabilities of Previous Window Previous Activities (PWPA) into the feature that leads to the best performance for streaming activity recognition [12]. Additionally, using a sensor dependency extension method and last state sensor method can improve the baseline techniques (SWMI and PWPA) when any event with missing labels are removed [13]. Similarly, using Online Stream Symbolisation (ODSS) can cluster a set of unlabelled routes in an efficient manner that is comparable to the equivalent clustering based on the Dynamic Time Warping approach (DTW) [16,17].

In this research, the prototype described in this paper uses the sensor-based windowing technique with a fixed size, which is the most commonly used segmentation method due to its simplicity of implementation [21,22,23] and is suitable for a prototype application applied on discrete binary motion detected by one resident only. To classify the data after having the raw data processed, suitable classification machine learning algorithm, for this purpose and for such kind of sensors, there are many approaches followed in the literature [2,3,9,10,12,24,25,26,27,28,29]. A Support Vector Machine technique (SVM) for activity recognition or detecting abnormal activities purposes can be used efficiently. Different types of approaches can be used; a one-class SVM that is trained on common normal activities can be used to filter out the activities that have a high level of probabilities to be normal, then both Kernel Nonlinear Regression (KNLR) and Maximum Likelihood Linear Regression (MLLR) can be used as a hybrid method to reduce the false-positive rate in an unsupervised manner, followed by calculating the performance efficiency of proposed algorithms by the Receiver Operating Characteristic (ROC) curve, which plots the detection rate against the false alarm rate, and finally calculating the Area under ROC curve values (AUC) with different algorithms and numbers of training traces, where it has been found that when the number of normal traces for training decreases, the performance of the two algorithms decreases accordingly [3,12].

Using the Hidden Markov Model approach (HMM) has been utilised intensively for pattern recognition in literature [2,9,10,24,25,26,27,28]. A dynamic HMM refinement procedure for temporal data clustering or having HMMs that can incrementally learn both the model’s parameters and structure are investigated in [25,28]. A novel sequence classification scheme by combining HMMs with the similarity-based paradigm that creates a representation space for sequences in which standard feature-based classification techniques can be used is adopted in [27]. Having a multiple classifier system that uses HMM is more accurate than those of single classifier systems [26]. Comparing the HMM approach with two other machine learning approaches, Naive Bayes Classifiers (NBC) and Conditional Random Fields (CRF) for recognition purposes using 11 datasets used from different types and sizes of environment by different numbers of residents indicate that when the number of residents is increased, the accuracy decreases. These rates represent that the Naive Bayes Classifier has the best accuracy among the other algorithms [2].

Many researchers have used the Naive Bayes theorem in their approaches due to its simplicity [20,29,30]. Efficient computational methods for selecting relevant features for Naive Bayes classification based on the sliding window method of stream mining with high predictive performance levels is identified in [20]. Additionally, a hybrid learning approach containing K-Means clustering and Naive Bayes classification is proposed in [29] in which the data is clustered into the corresponding group, then a Naive Bayes classifier is applied which resulted in low false alarm detection rate with an average below 0.5% while keeping the accuracy and detection rate on average higher than 99%.

## 3. Methodology

**Figure 1:**
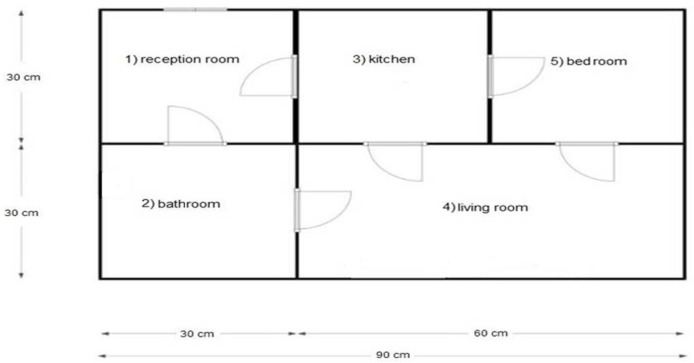
Blue print of the experiment prototype.

In a prototype that was developed as shown in Figure 1, the system observes the room-movement-sequence of the individuals under observation and the time spent in each room. When each observation is a sequence, probabilistic modelling and classification will be more difficult. As the length of the sequences will differ from one observation to another, standard pattern recognition techniques cannot be used directly. Sequence classification introduces extensive data sets [27].

The similarities between the observation sequences and classes can be computed using HMM. Each class represents a single HMM. The system chooses the maximum value of the similarity when making the classification decision. In the similarity-based approach, the classification decision can be made using all the sets of similarities between the observed sequence and all the classes. This method improves the classification performance [27].

However, the similarity-based approach has some problems such as high dimensionality of the resulting similarity space. There are two solutions for this problem. The first solution is building the similarity space using the total available patterns, and applying the standard dimensionality reduction technique. The second solution is choosing a small set of representatives [27].

In the HMM-based classification approach, the unknown sequence O is considered to follow the class with the highest likelihood. The classification rule as in: $Class\;\left(O\right)=Arg\;max_{i}P\left(O|\lambda_{i}\right)\;\;\;\;where\;\;\lambda_{i}:\;corresponds\;to\;the\;ith\;class$. This requires training certain HMM for each class (One Per Class (OPC)). In addition, one HMM can be trained for each training sequence (One Per Sequence (OPS)), and the considered unknown sequence O follows the class of the model, which has the highest likelihood. In this method, the classification rule becomes [27]: $Class\left(O\right)=ArgMax_{k}P\left(O|\lambda_{i}^{k}\right)$, $\lambda_{i}^{k}$: The HMM model trained on sequence $\Omega_{i}^{k}$, which follows class *k*.

P(O|λ) represents the probability that sequence O was generated by model λ. This quantity is called the likelihood and indicates the sequence similarity with the model. The similarity between two sequences $\Omega_{i}$ and $\Omega_{j}$ is $D_{i}$.

$D_{ij}=D\left(O_{i},O_{j}\right)=log\left(P\left(O_{i}|\lambda_{j}\right)\right)/T_{i}$$\lambda_{i}$: HMM is trained on sequence $O_{j}$ and $T_{i}$ is the length of sequence $O_{i}$. $1/T_{i}$ represents a normalisation factor using in account sequences of different length. The time-series data have a high dimensionality in most applications. Hence, a large number of features can be extracted to enable classification. Thus, applying classification techniques on the raw time series data is not useful nor practical. The raw data is replaced with a higher-level representation 184 to extract higher-order features to solve this challenge. The time-series data is then divided into a sequence of equal-sized windows. Many features can then be extracted from each window. The features vector produced from this division represents high-level representation for the raw time series data [26]. The features vectors are used to train a classifier and the classifier could be a Support Vector Machine (SVM), Neural Network (NN), rule induction, or any classifier. The state in the HMM is not directly observed. The state has a probability distribution over the possible output observations [26]. Time series data may be considered similar when they have a high correlation (share similar features). The models may be regarded as similar if the probability of the data generated by one model to the other model is high [28]. Given data *X* and model λ, which is trained from *X*, by applied Bayes theorem, the posterior probability of the model P(λ|X) equals [28]: P(λ|X)=P(λ)P(X|λ)P(X) P(X); the prior probability of the data. P(λ) is the prior probability of the model. P(X|λ) is the marginal likelihood of data. The goal is to choose the model that gives the highest marginal likelihood.

### 3.1. Adopted Algorithms

The selection of the algorithms is biased mainly by the problem we try to solve, the sensors and data type, and the prototype we adopted. The CNN is adopted mainly for its high accuracy in image detection and recognition problems, we used the ultrasonic array sensors in the living room to detect in real time the moving object within its scope and then translated the data set into an image that reflects the track of the motion pattern, with the image exported to CNN for both training and testing stages. Similarly, the Naive Bayes classifier, as a generative model, well suits the transition between rooms and works well with Boolean sensor data (PIR sensors) as it is less complex and therefore suits classification problems in real time. It also works well with the Hidden Markov Model transition between rooms matrix, as explained in this paper.

#### 3.1.1. Naive Bayes

Naive Bayes was selected as the prototype’s classifier of choice because it can be extremely fast compared to more sophisticated methods. It classifies new data concerning the highest probability of its belonging to a class. When the data set is small with many parameters, a Naive Bayes classifier is often the best choice. Naive Bayes classifiers are a family of probabilistic classifiers that apply Bayes’ theorem with strong independence assumptions between the features [31]. It has three types: Multinomial, Gaussian, and Bernoulli. The latter is adopted as the output of the classifier is Boolean (alarm on or off). In addition, a Naive Bayes is used when the model consists of scenarios that were not exactly in the training data thus, the simplicity in the implementation and updating on the arrival of new data, there being less required training data, high scalability, ability to make probabilistic predictions, ability to handle with continuous and discrete data, insensitivity towards irrelevant features, and ability to use it in real-time prediction. Moreover, Naive Bayes classifiers have high accuracy and speed on large data-sets. It is an accurate and reliable algorithm. These advantages led to the choice of Naive Bayes for the purpose of classification. For classification, the class ($c_{j}$) with the highest posterior probability is selected as the predicted class. For categorical features, the quantities $P_{r}\left(C=c_{j}\right)$ and $P_{r}\left(X=x_{i}|C=c_{j}\right)$ are estimated from the training data [20]. Depending on the probabilities that are evaluated on training data and the extracted features as (duration in each room, alternation of each room, the location of the person inside the room, the direction, and speed of movement) for two different classes that are used, the Naive Bayes theorem can classify the testing data into these two classes, normal or up normal.

Convolutional Neural Networks (CNNs): Convolutional Neural Networks are very effective in image recognition and classification. CNNs are based on four primary operations: Convolution, non-linearity (RelU), pooling or sub-sampling, and classification (fully connected layer). It helps to group unlabelled data (input data) according to similarities between the example inputs, and it can classify data (input data) when it has a labelled dataset to train on (training data). In addition to that, Neural networks can extract features that are fed to other algorithms for clustering and classification. Neural Networks can be used as components of larger machine-learning applications involving algorithms to improve the classification task.

The walking paths: When an individual’s location is detected using suitable sensors, their behaviour can be monitored and walking path detected. The motion pattern can then be analysed and classified. The walking path is considered a long term feature with observations and is very useful in motion and behaviour analysis [32]. Usually, a person performing legitimate activities do so at a moderate speed, and their walking path contain more regular segments. However, this is not always true for someone performing illegitimate activity [33]. During a particular time window (W), the co-variance matrix for the walking path segment is [32]:

$C:=\frac{1}{W}\sum \left(x_{t}-x\right){\left(x_{t}-x\right)}^{T}+\epsilon I$$x_{t}$, where the two dimension location ${\left(x_{t},y_{t}\right)}^{T}$ at time *t*, $x^{-}$ is the mean location in *W*, ϵ is the small positive constant, and *I* is the identity matrix.

#### 3.1.2. Convolutional Neural Network and Walking Paths

**Figure 2:**
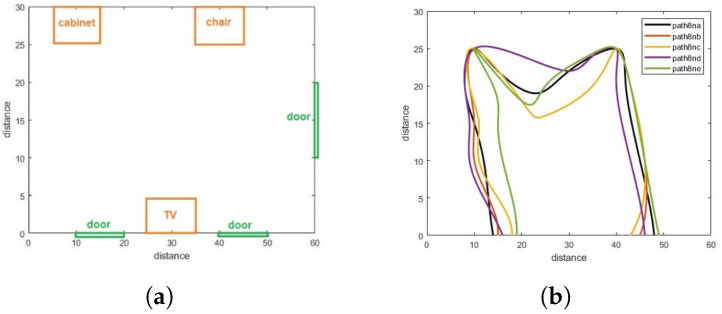
Walking paths in different observation for normal pattern. (**a**) The living room. (**b**) Multipaths for one pattern (normal).

**Figure 3:**
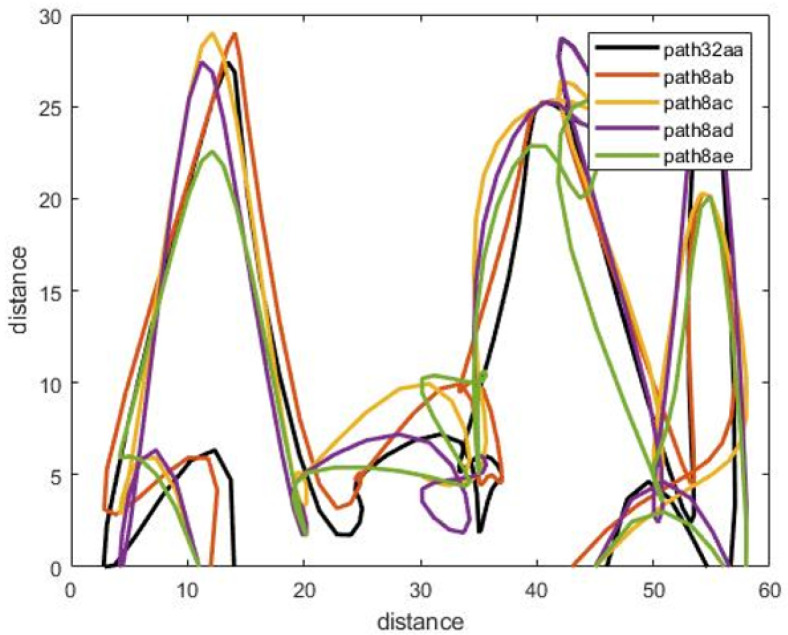
Walking paths in different observation for an abnormal pattern.

In the work articulated here, a CNN was applied to the walking paths image and divided into two classes: Normal and abnormal. When we monitor the walking paths for the person who lived in a certain space, such as in Figure 2a, we note that the walking paths in a different observation have a similar pattern as we illustrate in Figure 2 and Figure 3 (these paths represent the motion of the person in the living room which contains furniture in the following way, the data has been generated via the experiment prototype using synthetic motion trigger).

Walking paths for the abnormal motion can be represented in different ways, as illustrated in Figure 3.

The walking paths used represents the individual’s path while in the room. In total, a dataset of 180 images was constructed, consisting of normal and abnormal walking paths, and all dataset are gathered and uploaded to a data respiratory in this link: https://github.com/Ash83GH/Human-Activity-Recognition-Dataset (accessed on 17 December 2021).

**Figure 4:**
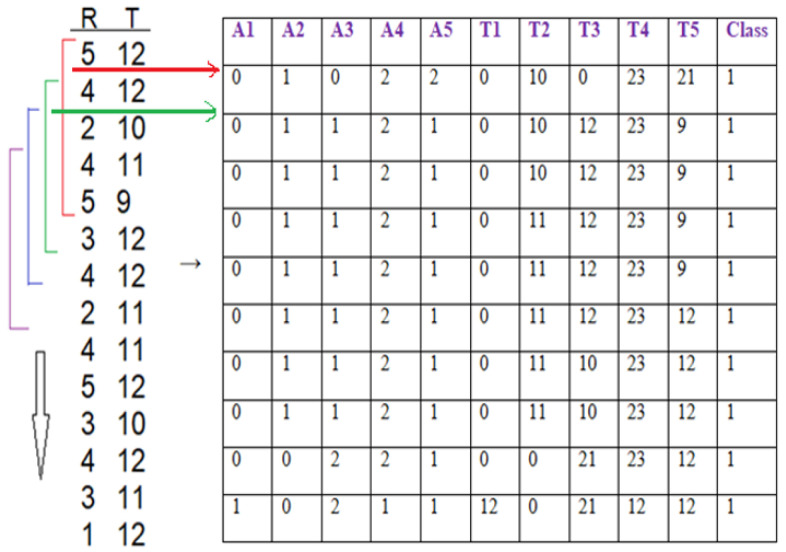
Data array and windowing logic.

**Figure 5:**
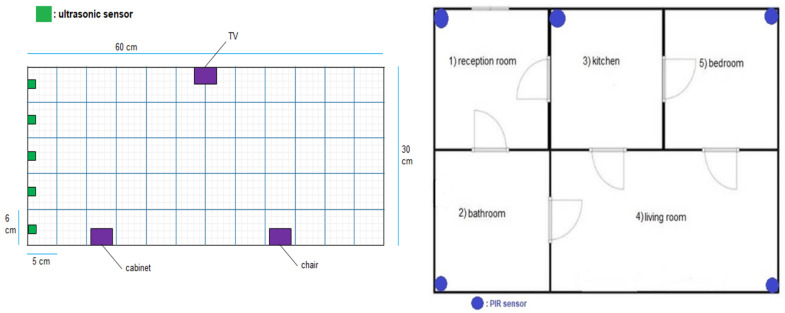
Prototype blueprint and living room ultrasonic sensor distribution.

Dataset: This system automatically learns the resident’s daily routine, including room-to-room transitions. Various algorithms are used to process the data from the sensors and extract the features representing normal mobility. Abnormal behaviour can be defined as any increase or decrease in daily activity. The PIR sensors in each room transmit a signal if any motion is detected. The observation O consists of the sensor ID (room number) and when the sensor detects the motion. The system facilitates binary classifications (0 = normal or 1 = abnormal). In the training phase, a set of motion pattern (training data) with labels (motion of the resident (normal) or intruder (abnormal)) are used to estimate a certain model. The unknown motion data (test data) is pre-processed and classified to the best model. The training data has been labelled based on the data collected mapped with the time slot of the day during weekly activity to get a sense of the individual’s daily activity pattern and decide whether the motion is normal or not. As seen in Figure 4, each training data contains some features about the motion, such as the alternation of each room (A1,A2,A3,A4,A5) and time duration that is spent in each room (T1,T2,T3,T4,T5) during a certain window size (in our project, we use a window size equal five). In addition, we have other features related to the person’s location inside the living room and the time spent in each location as shown in Figure 5. It is important to emphasise that the five ultrasonic sensors in the living room gives an approximate location of the moving person in the room. The accuracy of the position is estimated according to the grid division shown in Figure 5, the living room is not equipped with a PIR sensor as the detection is done via the set of ultrasonic sensors. Therefore, the walking path signature can be detected using the ultrasonic sensor and translated into a motion pattern image after being smoothed as shown in Figure 2 and Figure 3. Practically, we used a battery powered led connected to a stick. Prior to collecting the data, we decided what example motion patterns is considered normal or abnormal according to the positions of the doors and furniture. Then we moved the stick around to replicate the predefined motion patterns. The LED will trigger the PIR sensors, and the stick will trigger the ultrasonic sensors according to the position and deviation from the sensor. We used five ultrasonic sensors with a reflection accuracy of 5 cm and a 1-m range that helped us to visualise a grid of 5 times 12 units. The positions are reflected over a 2D grid matrix in MATLAB and the shaded squares that represent the movement are then smoothed after connecting lines between the centres of each square. Finally, a near accurate smooth motion is gained that reflects the walking paths.

### 3.2. Prototype and Tools

**Figure 6:**
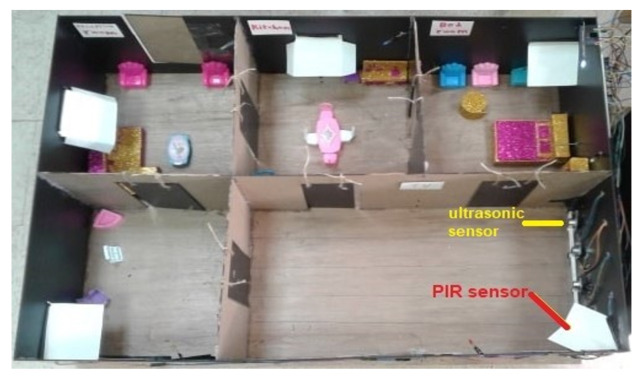
Prototype model.

The room sequence can be obtained using one PIR sensor in each room. The person’s location inside the room can be determined using an array from the ultrasonic sensors inside the room, as shown in Figure 5 and Figure 6.

Pre-processing: The collected data passes through feature extraction. The feature extraction requires that the data obtained from the sensors be digitised into time slices. The time slices may be constant or variable in length. This discretisation allows performing online (real-time) activity recognition and motion detection from digital motion sensors data in the building. Every sensor event in the sliding window will be classified based on the data encoded within it. Feature extraction also depends on the last state for each sensor event. Discretisation aims to divide the data into windows, suitable for the activity and motion pattern to be detected. Features are computed from each window and used for learning or testing [13].

Methods of windowing streaming data. (1) Activity-based windowing [23]: The streaming data is divided into windows at the start of the first motion detected and any activity changes. Some features can be extracted from every window. (2) Time-based windowing: Streaming data are divided into fixed time windows. It is the simplest method in the windowing process, and it is appropriate for continuous data sensor and motion detection. Classification errors using this method come from an inappropriate window size. When the window’s length is too small, the information in the window may be insufficient for making a decision. When the length is too long, information concerning multiple activities may be embedded within a single window frame. (3) Sensor-based windowing: Data are divided into windows of an equal number of streaming data from sensor events. The resulting windows size differs from one to another.

**Figure 7:**
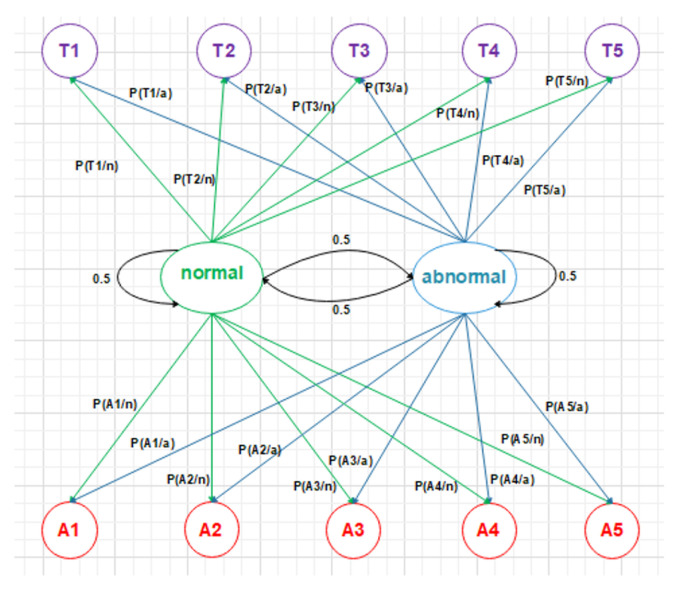
State transition probability.

There are different ways to extract features from the sequence of sensor data concerning an individual. Many features can be extracted from the sensors, such as room ID, sequence of rooms visited, time spent in each room, repetition of room visiting sequence, location of the individual within the room, and speed of individual’s movement [13]. (1) Baseline method: When the sensor data window is defined, the window can be transformed into a feature vector. The sensor-based windowing method suffers from problems such as the possibility of the window containing sensor data widely separated in time. These issues can be avoided using a time-based weighting scheme that considers the relative difference in each sensor’s triggering. (2) Sensor dependency method [13]: Sometimes, features from the data of the sensor will make some expectation to the next sensor data. For example, if the person exists in the bedroom in the morning, the next activity is often going to the bathroom. In addition, dependence between sensors is defined as the chance of occurring two sensor events consecutively in the same window. This approach is known as Sensor Window Mutual Information (SWMI) for future reference. (3) Sensor dependency extension method [13]: Mutual information of two sensors depends on the order of the pair of sensors in the entire data stream. To perform a particular activity in a specific room, a person might have different route options. For example, in the prototype, the person can get to a room using five different paths: S1→S2→S4→S5orS1→S3→S5. The most widely used machine learning algorithms for detecting human motion patterns are the Naive Bayes and neural networks [11]. In the training phase, a set of motion pattern (training data) with labels (movement of the homeowner (normal) or the motions of the intruder (abnormal)) are used to estimate a particular model. The unknown motion data (test data) will be pre-processed and classified to the best model, in the Naive Bayes case, the conditional probabilities of the features and observations are then estimated to estimate the maximum likelihood as shown in Figure 7. Arduino Mega connected to the PIR sensors and interfaced to a PC with Intel core 7 CPU and 4 GB RAMs have been used to collect the data. MATLAB installed over the PC for pre-processing and for CNN training purposes. Finally, we used a simple buzzer connected to Arduino Mega that is interfaced to a PC. The classification mechanism implemented in the PC and the outcome (NORMAL or ABNORMAL) is decided and finally, if ABNORMAL, a trigger signal is sent to Arduino MEGA to activate a buzzer sound.

## 4. Results and Discussion

The smart alarm system’s performance is measured by the detection rate and the false alarm rate. The detection is the ratio of correctly detected suspicious motions to the total number of real suspicious motions. The false alarm rate is the ratio of the number of normal motions that are incorrectly detected as a suspicious motion to the total number of real normal motions [3].

False alarm rate = FP/(FP + TP)

A smart alarm system’s performance must have a high detection rate and a low false alarm rate [3]. The following results were obtained when considering 25 normal motions and 25 abnormal motions.

**Figure 8:**
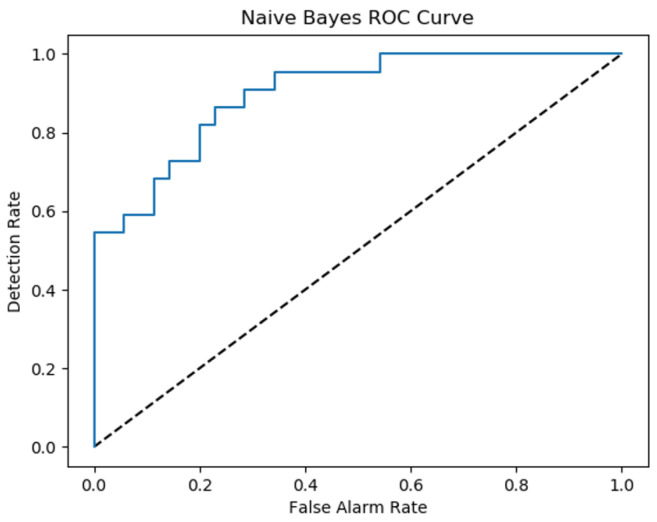
Walking paths in different observation.

Figure 8 shows ROC curve for the Naive Bayes depending on applied experiments.

The motion represents the trajectory of the individual’s position with respect to time. At each position, the speed and direction will change smoothly and gradually. The motion pattern can be defined as regularity in space change or time change or any other relation between motion features. If the individual’s movement is compared to itself over time, then we can define and detect the anomaly behaviour within a short period of time. The motion’s pattern is considered constant when a moving person has motion features that are constant over time [33].

The temporal movement features include how often, how long, when, and how regularly a person moves. The principal measurements in the time features are a moment and a time interval. The moment represents the time at which a moving person exists. The moment is considered primary motion features. The time interval is the time difference between two moments and is regarded as a derived motion feature. The spatial movement features [33] include how far, where, and in which direction a person is moving. The principal measurement in the spatial features is a spatial position in which a moving person reaches. The spatial path or walking path represents the ordered list of measured spatial positions. The spatial path is considered primary motion features. The distance between two positions on a walking path represents the straight line’s length between two places. The distance travelled along the walking path is determined by counting the number of grid squares that are continuously connected and reflecting each grid to the positions by linear interpolation and adding all spatial distance between every two positions. The spatial and temporal motion features [33], with each location recorded at a particular time, can be combined into a single feature that can be called a spatio-temporal location *P*(*t*) = (*x*(*t*), *y*(*t*)). When there is a sequence of locations ($P(t_{1}$), $P(t_{2})$, …, $P(t_{n})$), the speed of the motion can be computed between two locations V=ΔP/Δt, and the acceleration can also be calculated (the change of speed) A=ΔV/Δt. The speed and acceleration are considered derived features. Instantaneous feature: Location, time moment for each location; short-term feature: Direction of movement, speed, time duration in each location, and alternation of each location; long-term feature: Walking path and travel distance.

**Figure 9:**
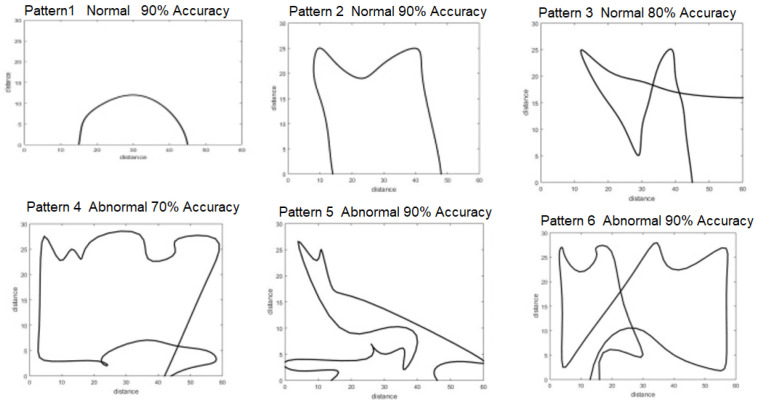
Different walking path scenarios with accuracy estimation.

**Figure 10:**
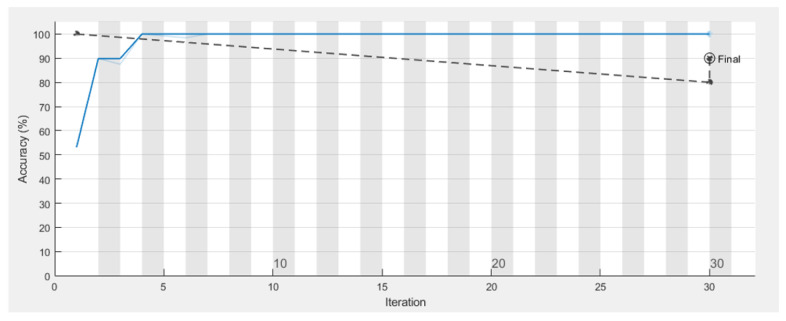
Accuracy rate of CNN during the training phase.

**Figure 11:**
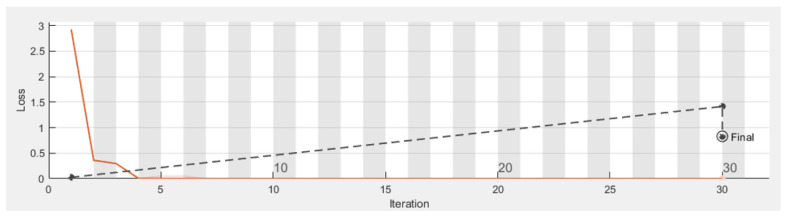
Loss rate of CNN during training phase.

Figure 9 shows the walking paths obtained using the experimental prototype for different scenarios from different patterns of walking paths for normal and abnormal motion to be evaluated using CNN along with detection accuracy for each pattern. Using TensorFlow, it can be seen that the detection accuracy of the CNN following the training phase is accurate enough as shown in Figure 10, and this is also reflected in the loss rate shown in Figure 11; the training phase lasted for 30 iterations until it converges for a stable low enough loss rate and accuracy rate.

**Figure 12:**
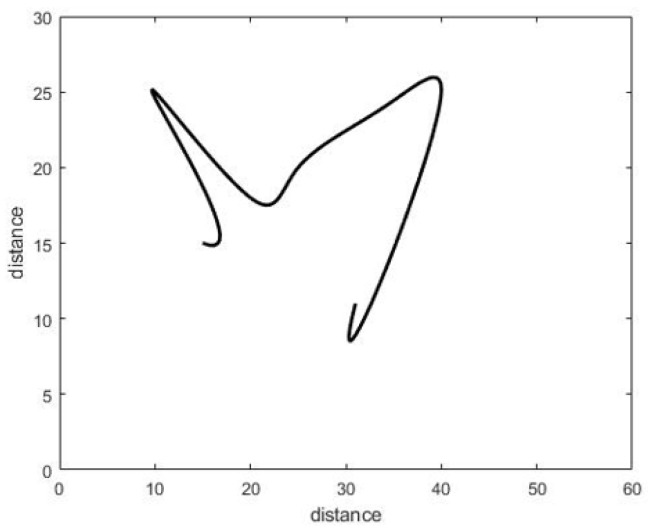
Partial normal path.

**Figure 13:**
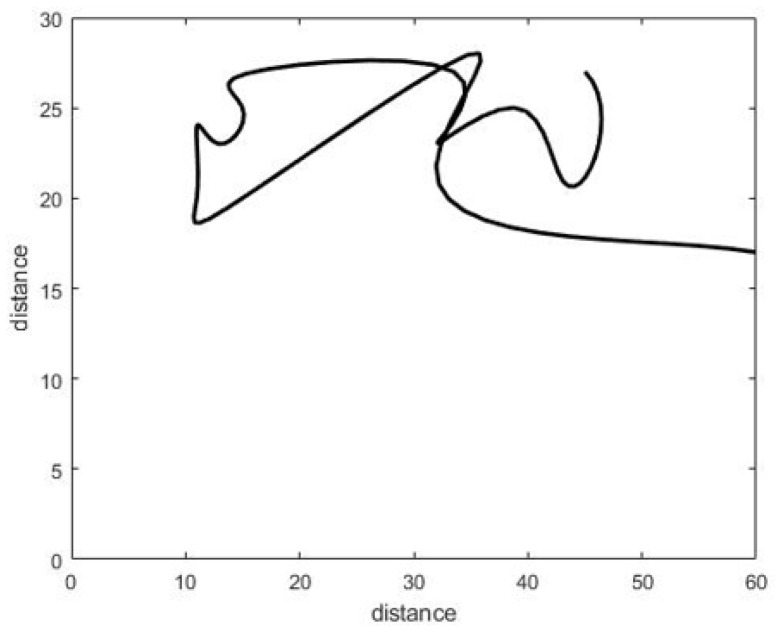
Single Partial abnormal path extracted using the prototype.

We use the complete walking path from the moment of entry to the moment of exit from the the room in the training data and in the test experiment. We will try to test the walking paths that represent part of the motion not necessarily from the moment of entry to the moment of exit, as illustrated in Figure 12 and Figure 13. The training data remain the same (complete walking paths).

**Figure 14:**
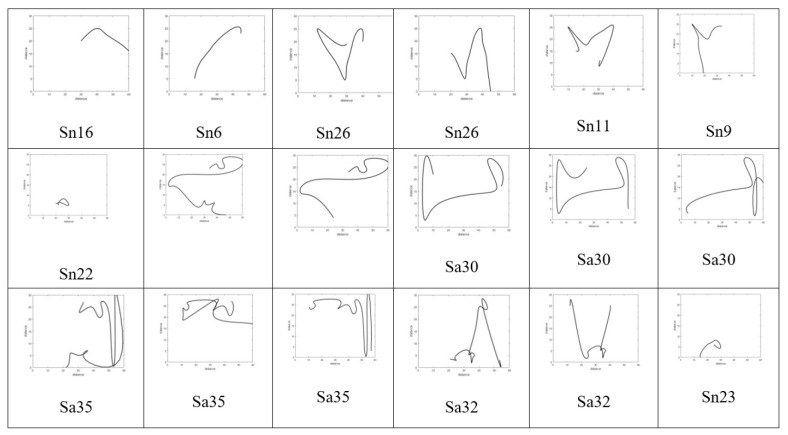
Multiple Partial abnormal paths for different scenarios extracted using the prototype.

**Figure 15:**
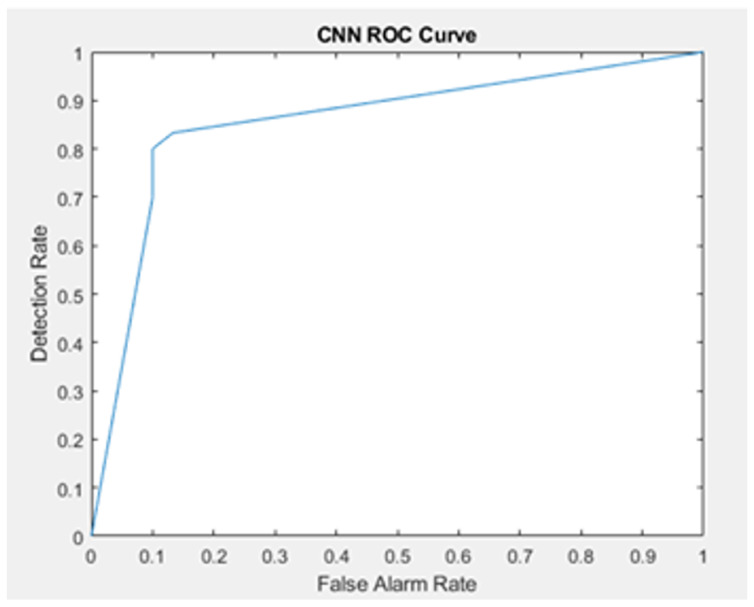
Different scenarios of partial paths receiver operating characteristic (ROC) curve.

The complete walking path is estimated starting from room entry till the exit, and this is measured for both the training and testing data set. In reality, there is a need to detect the anomalies in near real-time period, this means that there is no time to wait until the entire validation data set being stored from the entrance to exit to reflect a full walking path patters. Therefore, we test the walking path representing part of the motion, not necessarily from the moment of entry to the moment of exit, as illustrated in Figure 12 and Figure 13. The training data remains the same (complete walking paths). Following this, testing using groups of uncompleted paths, an accuracy of 77.78% was obtained after applying the partial walking paths in Figure 14 using CNN in MATLAB. The CNN ROC curve is then estimated as shown in Figure 15.

**Figure 16:**
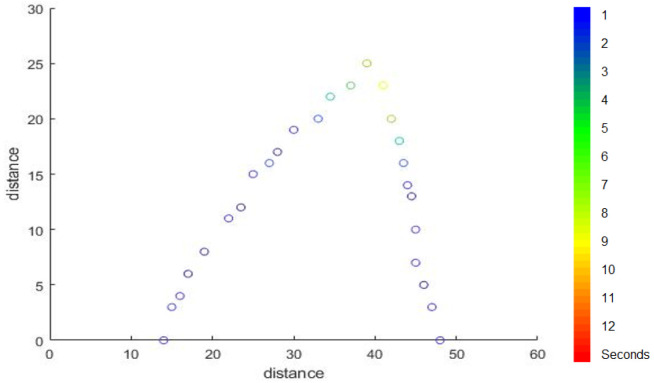
Delay colour gradient.

If we want to indicate the amount of time that is spent in every location in the walking path figure, we can use the gradient colours to this purpose. We use a light colour in the locations showing that the person spends a long time in it and used a dark colour in the location where the person spends a short time, as shown in Figure 16.

**Figure 17:**
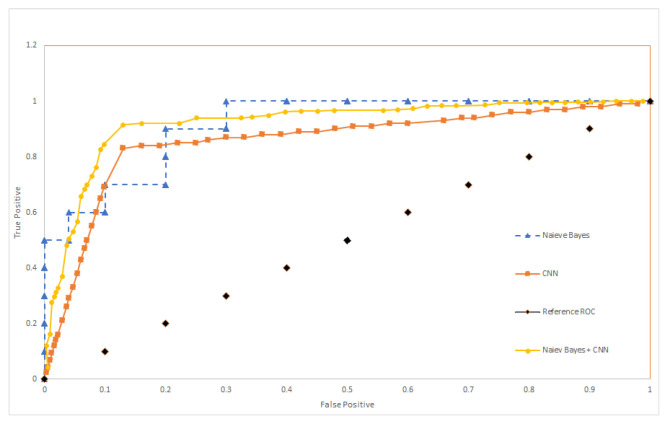
ROC comparison between different algorithms.

To compare the performance of the aforementioned algorithms (Naive Bayes, CNN) and to have an insight into the performance of the combined algorithm (Naive Bayes + CNN). ROC curves are plotted in Figure 17. The reference ROC is used to tell if the algorithm is able to discriminate between classes or not, as long as the ROC curves exceed this threshold, it can classify successfully (with certain accuracy). To better understand the performance, we estimate the Area under Curve (AUC) for each algorithm and compare it against other AUC values presented in the related study section. The AUC values is shown in Table 1. The combined CNN and Naive Bayes outperforms the other algorithms for the setup we have in the experiment.

## 5. Motion Pattern Description between Rooms

For this experiment, we used a simple Arduino Uno micro-controller to collect the PIR sensor triggers and export it to MATLAB for further processing, the full PIR raw data, and a features data set is uploaded to https://github.com/Ash83GH/Human-Activity-Recognition-Dataset (accessed on 17 December 2021). The stay in the room is computed through the time difference between the two observations (ts2 − ts1). Where ts1 represents the moment when the person enters the room and ts2 represents the time they enter the next room. It is assumed that the motion is checked every Δ*t*, where Δ*t* is one hour. Maybe a specific stay will overlap with other intervals in the system. For example, if a stay duration in a particular room is 1.5 h, which starts at ts1 = 02:00 and finishes at ts2 = 03:30, distributed between two intervals, one hour in the interval (2–3) and 30 min in the interval (3–4) [23]. The second step computes the total stay time in a room during a specific time interval. This total stay is computed using the following equation [34]: $TS_{i}=\sum_{k=1}^{ns^{i}}s_{k}^{i}\left(t,\Delta t\right)$ where $s_{k}^{i}\left(t,\Delta t\right)$ is the *k*th stay in room *i* and $ns^{i}$ is the number of stays in the room *i* during the time duration from *t* to (t+Δt).

The third step computes a self-transition or stay probability. The stay probability $P^{i,i}\left(t,\Delta t\right)$ in room *i* in a specific time interval equals the probability of exiting the person in room *i* during interval *t* to (t+Δt) [34]. The following equation represents the stay probability:

$$P^{ii}\left(t,\Delta t\right)=\frac{TS_{i}\left(t,\Delta t\right)}{\sum_{j=1}^{r}Ts^{j}\left(t,\Delta t\right)}=\frac{TS_{i}\left(t,\Delta t\right)}{\Delta t}$$

where *r* is the number of rooms in the house

**Figure 18:**
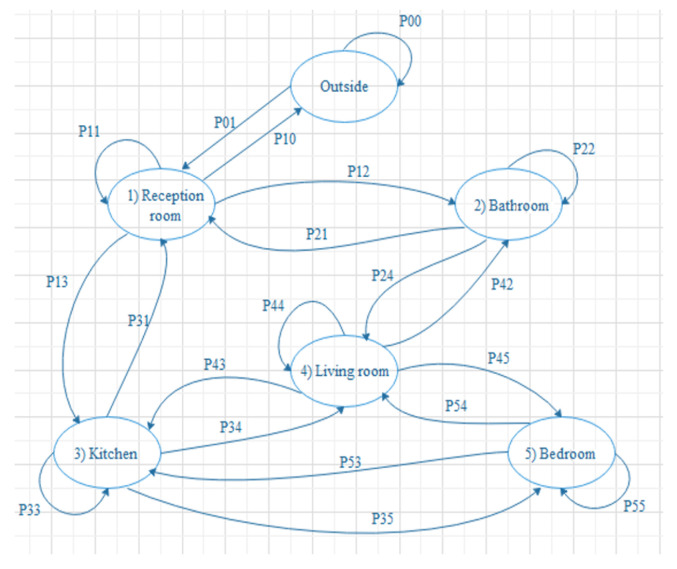
Transition states between rooms.

The fourth step computes the transition probability. The transition probability $P^{i,j}\left(t,\Delta t\right)$ during a specific time interval *t* to (t+Δt) equals the probability of transition for a person from room *i* to room *j* during the interval *t* to (t+Δt). Figure 18 shows both the stay and transition probabilities of the experiment. The following equation represents the transition probability [34]:

$$P^{ij}\left(t,\Delta t\right)=\left[1-P^{i,i}\left(t,\Delta t\right)\right]\times \frac{M^{i,j}\left(t,\Delta t\right)}{\sum_{k=1}^{r}M^{i,k}\left(t,\Delta t\right)}$$

where $M^{i,j}\left(t,\Delta t\right)$ is the number of transitions from room *i* to room *j* during the time duration *t* to (t+Δt), (*j* ≠ *i*) and $\sum_{j=1}^{r}P^{ij}\left(t,\Delta t\right)$.

We will introduce a cumulative dimension to compute the total number of observations within a specific interval. This dimension is called the global activity (AG). It represents the level of activity in the house. The following equation computes the AG:

$$AG\left(t,\Delta t\right)=\frac{1}{\Delta t}\times \#\left(O\left(t,\Delta t\right)\right)$$

where #(O(t,Δt)) is the number of items in the group from the observations received from the sensors during the time interval *t* to (t+Δt). Another dimension represents the total number of times a sensor was activated to a transition during a specific interval except for the self-transitions. This dimension is called the inter-room activity (AE). This dimension indicates how a person moves between rooms during a particular time interval. This dimension detects the transitions between different rooms, while the global activity dimension detects the activity that often is in a single room. The following equation computes the AE dimension:

$$AE\left(t,\Delta t\right)=\frac{1}{\Delta t}\times \#\left(M\left(t,\Delta t\right)\right)-\sum_{i=1}^{r}\#\left(M^{i,i}\left(t,\Delta t\right)\right)$$

where (#(M(t,Δt)) is the number of items in the group of all the transitions within the time interval *t* to (t+Δt).

And $\#\left(M^{i.i}\left(t,\Delta t\right)\right)$ is the number of items in the group of the self-transitions in room *i*.

Furthermore, another dimension represents the total number of self-transitions in a room during a specific interval. This dimension is called the intra-room activity (AA). This dimension computes the total number of the received observations in a room during a particular interval and illustrates how the person is active in every room. The following equation calculates the AA dimension [34]:

$$AA^{i}\left(t,\Delta t\right)=\frac{1}{\Delta t}\times \#\left(O^{i}\left(t,\Delta t\right)\right)$$

where $\#\left(O^{i}\left(t,\Delta t\right)\right)$ is the number of items in the group of the received observations in room *i* during the time interval *t* to (t+Δt)

The final dimension is the intra-room Continuous Stay (CS). This dimension indicates the longest continuous stay in every room. A continuous stay is a sequence of sequential stays or a single long stay in the same room. The following equation computes this dimension [34]:

$$CS^{i}=\sum_{k=1}^{u}d\left(s_{k}^{i}\right)$$

where $d(s_{k}^{i})$ is the interval of the *k*th stay in room *i* and *u* is the number of sequential stays in room *i*.

Transition matrix:

$$\left[\begin{array}{cccccc} P00 & P01 & 0 & 0 & 0 & 0\\ P10 & P11 & P12 & P13 & 0 & 0\\ 0 & P21 & P22 & 0 & P24 & 0\\ 0 & P31 & 0 & P33 & P34 & P35\\ 0 & 0 & P42 & P43 & P44 & P45\\ 0 & 0 & P52 & P53 & P54 & P55 \end{array}\right]$$

## 6. Conclusions

This paper proposes an approach for classification detected motion patterns using PIR, ultrasonic sensors. The features are extracted from detected motion according to spatio-temporal parameters that are reflected over the blueprint design of the building. Then Naive Bays algorithm is used to classify the detected motion pattern to be normal or abnormal motion depending on training data collected previously. In the final stage, the CNN approach is also used for classification purposes combined with Naive Bays for a better accuracy and detection rate, CNN is applied over walking path patters inside the living room. Our approach’s primary advantage is that it can achieve a better detection rate and lower false alarm rate. We demonstrated our hybrid approach’s effectiveness (Naive Bays, CNN) using real data collected from sensors distributed in different rooms in the proposed building. A potential limitation of our approach is that there is interference in ultrasonic sensor signals. Thus, it affects the detected walking path and gives a false alarm. To solve this problem, we used a 10:1 scaled prototype with better control over the sensor range so as to avoid the scattering of the sensor beams. Another limitation is that, in this paper, we assumed for one resident in the proposed small building. Having hybrid machine learning algorithms such as Naive Bayes that is combined with CNN boosts the accuracy of the detection rate by having another check of the motion subset, which also increases the number of features to better describe the entire motion or partial motion patterns.

## Figures and Tables

**Table 1:** AUC values for performance comparison.

Algorithm	AUC
CNN	0.835
Naive Bayes	0.521
CNN + Naive Bayes	0.909
OneSVM	0.689
SVM + KNLR	0.739
SVM + MLLR	0.706

## Data Availability

Data used for carrying out the experiments in this research is generated out of an experimental prototype and publicly available via this link: https://github.com/Ash83GH/Human-Activity-Recognition-Dataset (accessed on 17 December 2021).
